# Draft Genomic Sequences of Four Pseudomonas spp. and a *Xanthomonas* sp. from Cranberry Stem Galls

**DOI:** 10.1128/mra.00999-21

**Published:** 2022-03-01

**Authors:** Brian Graham, Tawny Castañeda, Alisha Harrison, Scott Soby

**Affiliations:** a Biomedical Sciences Program, College of Graduate Studies, Midwestern University, Glendale, Arizona, USA; b Arizona College of Osteopathic Medicine, Midwestern University, Glendale, Arizona, USA; c College of Veterinary Medicine, Midwestern University, Glendale, Arizona, USA; SIPBS, University of Strathclyde

## Abstract

Four Pseudomonas spp. and Xanthomonas arboricola were isolated from cranberry stem galls in Carver, MA, and taxonomically assigned at the genus level based on the 16S rRNA sequence and phenotypes. X. arboricola had not been associated previously with cranberry stem galls or any cranberry disease.

## ANNOUNCEMENT

Stem galls on cranberry plants (Vaccinium macrocarpon Ait.) result from hyperplasia and hypertrophy in response to the production of phytohormones by bacteria that have invaded stem tissue, usually following mechanical or frost injury to the epidermis (1). Galls are relatively common in commercial cranberry operations in regions with especially cold winters and are likely the result of mixed infections, of which some members produce indole-3-acetic acid (IAA) (2). Although infrequently observed in Massachusetts, galls can girdle the stem, resulting in the death of meristems and fruit-producing organs. Bacteria were isolated from multiple stem galls on several plants in a commercial cranberry bog in Carver, MA, following the severe 2015 winter by spreading surface-sterilized gall tissue on nonselective media. Five of the isolates were transferred to King’s medium B (KMB) agar containing 50 μg · mL^−1^ each of cycloheximide and ampicillin, incubated at 26°C for 24 to 48 h, colony purified 3 times, and stored at −80°C in 34% glycerol. Four isolates were placed in the genus Pseudomonas and one in *Xanthomonas* by 16S rRNA gene sequences amplified with 27F and 1525R primers, using BLAST (3) within the NCBI nucleotide database. Isolates from frozen storage were recovered on KMB agar, and then populations were inoculated into overnight KMB broth cultures for genomic DNA isolation with a DNeasy blood and tissue kit (Qiagen). Illumina-compatible genomic DNA (gDNA) libraries were generated with a Kapa Biosystem Hyperplus library preparation kit (KK8514). DNA was enzymatically sheared to approximately 500-bp fragments, end repaired, and A-tailed. Illumina-compatible adapters with unique indexes (Integrated DNA Technologies [IDT] number 00989130v2) were individually ligated to each sample, cleaned using pure beads (Kapa Biosciences; KK8002), and amplified with a HiFi enzyme (KK2502). Each library was analyzed for fragment size (Agilent Tapestation) and quantified by quantitative PCR (qPCR) (Kapa library quantification kit, KK4835; Thermo Fisher Scientific, Quantstudio 5) before multiplex pooling and Illumina MiSeq sequencing on a 2 × 250-bp flow cell. The assembly of raw reads was done by Unicycler v0.4.8 (4) and polished with Pilon v1.23 (5) within the PATRIC Comprehensive Genome Analysis pipeline v3.6.12 using default settings (http://patricbrc.org) (6) (Table 1), which includes Trim Galore v0.4.0 (https://www.bioinformatics.babraham.ac.uk/projects/trim_galore/) (7) for adapter trimming and quality control. Genome sequences were annotated using RASTtk (8) as part of the PATRIC pipeline. Using the Type (Strain) Genome Server (9), isolates were placed within Pseudomonas syringae (MWU16-30316), Pseudomonas putida (MWU16-30317), or Pseudomonas fluorescens subgroups P. fluorescens (MWU16-30323) and Pseudomonas koreensis (MWU16-30322). MWU16-30325 is most closely related to X. arboricola pv. *pruni*. RAST annotation indicates that MWU16-30322, MWU16-30316, and MWU16-30325 contain the gene (*ina*) required for the ice nucleation protein that is associated with frost damage (10, 11) and that *Xanthomonas* sp. MWU16-30325 in addition contains genes for assembly and translocation of xanthan (12).

**TABLE 1:** Data summary

Isolate	Assigned taxon	Genome size (bp)	No. of contigs	*N*_50_ contig size (bp)	Coverage (×)	G+C content (%)	Total length of reads (bp)	No. of reads	BioSample accession no.	GenBank accession no.	SRA accession no.	No. of coding sequences	No. of tRNA/rRNA genes
MWU16-30322 (Erika 2)	Pseudomonas nov. sp.	6,193,752	37	428,342	362	60.41	2,241,274,727	4,830,691	SAMN21542436	JAIWYL000000000	SRX12391655	5,709	63/2
MWU16-30316 (Erika 3)	Pseudomonas nov. sp.	5,994,666	25	858,102	301	59.17	1,806,931,759	3,033,833	SAMN21542437	JAIZAZ000000000	SRX12300885	5,350	55/4
MWU16-30323 (Erika 4)	Pseudomonas nov. sp.	6,669,642	112	205,587	164	60.86	1,090,791,137	2,442,946	SAMN21542439	JAIWYM000000000	SRX12391656	6,149	46/2
MWU16-30317 (Erika 5)	Pseudomonas nov. sp.	6,473,724	37	904,938	1668	61.39	2,744,746,383	5,223,120	SAMN21542440	JAIWJA000000000	SRX12300886	5,888	58/3
MWU16-30325 (Erika 7)	*Xanthomonas* sp.	4,811,759	13	1,303,235	288	65.89	1,384,143,382	2,899,649	SAMN21542459	JAIWYN000000000	SRX12391657	4,293	51/3

### Data availability.

This whole-genome shotgun project has been deposited at DDBJ/EMBL/GenBank BioProject PRJNA765055 under the accession numbers JAIWYL000000000 (MWU16-30322), JAIZAZ000000000 (MWU16-30316), JAIWYM000000000 (MWU16-30323), JAIWJA000000000 (MWU16-30317), and JAIWYN000000000 (MWU16-30325). The versions described in this paper are the first versions, JAIWYL000000000.1, JAIZAZ000000000.1, JAIWYM000000000.1, JAIWJA000000000.1, and JAIWYN000000000.1, respectively. Links to SRA accessions are provided in Table 1. RASTtk annotations are available under open license at Zenodo (https://zenodo.org/record/5949069#.Yf1TcfnMKUl).
